# Galectin-3: Roles in Neurodevelopment, Neuroinflammation, and Behavior

**DOI:** 10.3390/biom10050798

**Published:** 2020-05-21

**Authors:** Ivan Srejovic, Dragica Selakovic, Nemanja Jovicic, Vladimir Jakovljević, Miodrag L. Lukic, Gvozden Rosic

**Affiliations:** 1 Department of Physiology, Faculty of Medical Sciences, University of Kragujevac, Svetozara Markovica 69, 34000 Kragujevac Serbia; ivan_srejovic@hotmail.com (I.S.); dragica984@gmail.com (D.S.); drvladakgbg@yahoo.com (V.J.); 2 Department of Histology and Embryology, Faculty of Medical Sciences, University of Kragujevac, Svetozara Markovica 69, 34000 Kragujevac, Serbia; nemanjajovicic.kg@gmail.com; 3 Department of Human Pathology, 1st Moscow State Medical University IM Sechenov, 119146 Moscow, Russia; 4 Department of Physiology—Molecular Medicine Unit, Faculty of Medical Sciences, University of Kragujevac, Svetozara Markovica 69, 34000 Kragujevac, Serbia

**Keywords:** Galectin-3, neurodevelopment, neuroinflammation, behavior

## Abstract

There is a plethora of evidence to suggest that Galectin-3 plays an important role in normal functions of mammalian cells, as well as in different pathogenic conditions. This review highlights recent data published by researchers, including our own team, on roles of Galectin-3 in the nervous system. Here, we discuss the roles of Galectin-3 in brain development, its roles in glial cells, as well as the interactions of glial cells with other neural and invading cells in pathological conditions. Galectin-3 plays an important role in the pathogenesis of neuroinflammatory and neurodegenerative disorders, such as multiple sclerosis, Alzheimer’s disease, Parkinson’s disease, and Huntington’s disease. On the other hand, there is also evidence of the protective role of Galectin-3 due to its anti-apoptotic effect in target cells. Interestingly, genetic deletion of Galectin-3 affects behavioral patterns in maturing and adult mice. The results reviewed in this paper and recent development of highly specific inhibitors suggests that Galectin-3 may be an important therapeutic target in pathological conditions including the disorders of the central nervous system.

## 1. Structure of Galectin Proteins

**Figure 1:**
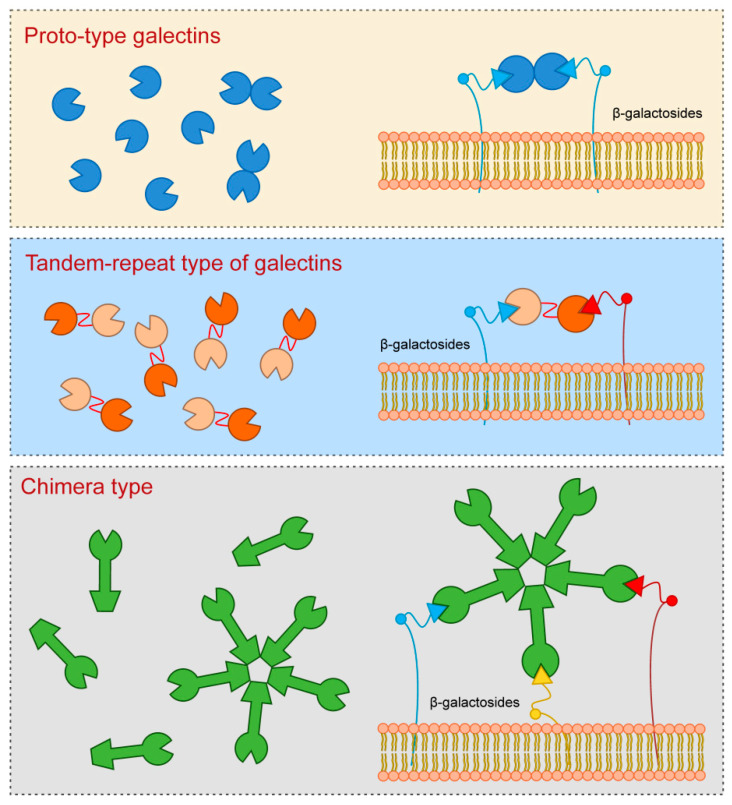
Galectins classification (according to their structures).

Galectins are a widespread group of proteins, both in different cells and tissues, and across diverse metazoan species [1,2]. They contain evolutionarily highly conserved carbohydrate-recognition domains consisting of approximately 130 amino-acids which bind β-galactose in glycoconjugates. In mammalian tissues, the existence of 15 galectins has been established so far, which are classified into three groups according to their structures [3,4,5] (Figure 1).

Proto-type galectins that have a single carbohydrate-recognition domain (CRD), which are also the most numerous subgroup of galectins, include Galectin-1, Galectin-2, Galectin-5, Galectin-7, Galectin-10, Galectin-11, Galectin-13, Galectin-14, and Galectin-15.

Tandem-repeat type of galectins which contain two similar CRD, include Galectin-4, Galectin-6, Galectin-8, Galectin-9, and Galectin-12.

## 2. Structure and Function of Galectin-3

Galectin-3, which is a sole representative of chimera type, contains one CRD which is linked to the N-terminal domain that allows oligomerization resulting in formation of pentamers. Specifically, upon interaction of Galectin-3 monomers with glycoproteins or glycolipids they interconnect to form pentameric complex by their N-terminal domains (Figure 1).

Galectin-3 is a protein with approximate molecular weight of 31 kDa, first recognized in murine immune cells, and thereafter found in a variety of normal and tumor cells [3,6,7]. The structure of Galectin-3 is unique among all vertebrate galectins, consisting of two structurally specific domains: N-terminal domain and C-terminal CRD [8]. N-terminal domain usually contains up to 150 amino acid residues, depending on the species, built up of nine repetitive sequences rich in proline, glycine, tyrosine and glutamine [3,6,9]. N-terminal domain carries sites for phosphorylation and other determinants involved in regulation of galectin secretion [10,11,12]. C-terminal CRD, consisting of about 135 amino acid residues, and determines the molecule as a galectin family member. CRD is connected to a collagen-like sequence, assembled of proline, glycine and tyrosine tandem repeats [13].

In adult humans Galectin-3 is present in many different types of cells and tissues. During the first trimester of human embryo development, Galectin-3 is mainly expressed in epithelia, such as lining epithelium of the respiratory system and digestive tract, urothelium, skin, as well as in myocardium, liver and chondrocytes [14]. In adults Galectin-3 is also found in various immune cells, except resting lymphocytes [3,8,15]. Apart from physiological functions in a variety of biological processes such as cell adhesion, cell activation, cell growth and differentiation, cell cycle, and apoptosis, Galectin-3 also has pivotal roles in cell to cell interactions [8,16,17,18].

Galectin-3 synthesis takes place on free ribosomes in the cytoplasm. It can be found in nucleus, on the cell surface and in the extracellular space [19,20]. Given that Galectin-3, as all galectins, lacks a signal sequence that would guide its translocation to endoplasmic reticulum and further enable classical secretory pathway, the secretion of Galectin-3 takes place in a non-classical fashion [20]. Depending on the cell type, Galectin-3 is found in exosomes or microvescicles [21,22,23]. It has recently been shown that endosomal sorting complex required for transport (ESCRT) machinery plays a crucial role in Galectin-3 transport to the extracellular matrix. Specifically, the ESCRT-I component Tsg101 binds to highly conserved P(S/T)AP motif located to N-terminal domain of Galectin-3 allowing the packing of Galectin-3 into endosomes [24]. Biological effects of Galectin-3 are largely determined by its cellular localization, specific tissue, or specific pathological condition. The most exhaustively studied role of Galectin-3 concerns the regulation of inflammatory processes. Pathogenesis of xenobiotic induced Primary Biliary Cholangitis (PBC) indicated protective role of Galectin-3, since PBC in Galectin-3 knock-out (KO) C57Bl/6 mice showed enhanced liver infiltration with CD8+ T lymphocytes followed by augmented bile duct damage, liver fibrosis, serological level of PDC-E2 (E2 component of the pyruvate dehydrogenase complex—common auto-antigen in PBC) specific IgA and increased AST/ALT ratio [25]. Conversely, recent study by Arsenijevic and colleagues pointed out detrimental role of Galectin-3 in PBC with infectious etiology [26]. In *Novosphingobium aromaticivorans* (*N. aromaticivorans*) induced PBC, Galectin-3 deletion had anti-inflammatory role, due to the decreased activation of dendritic cells and macrophages in Galectin-3 KO C57Bl/6 mice. Furthermore, we showed that in mouse experimental model of autoimmune myocarditis Galectin-3 had a protective role on disease development [27]. In myosin peptide-induced experimental autoimmune myocarditis (EAM) on C57Bl/6 mice, Galectin-3 KO mice developed more severe myocardial inflammation and more conspicuous hypertrophy, due to the accumulation of T helper type 2 (Th2) cells and expansion of type 2 inflammation in the hearts of otherwise predominantly Th1 C57Bl/6 mice.

## 3. Galectin-3 Ligands and Pattern Recognition Receptors in the CNS

There are numerous biological ligands for Galectin-3 which, as with galectin 3, have a diverse distribution, both in the cell and in the extracellular space. Intracelular Galectin-3 ligands are gemin 4, Bcl-2, nucling, synexin, and β-catenin, while in extracellular space Galectin-3 binds to glycoproteins which contain β-galactoside, such as laminin, fibronectin, CD29, CD66, α1β1 integrin, and Galectin-3-binding protein [3,6,28].

Extracellular Galectin-3 binds to ligand via CRD and is involved in inflammation, cell to cell and cell to matrix interaction and function as Advanced glycation end products (AGE) receptor [29]. Galectin-3 N-terminal domain binds its ligand to protect from apoptosis [30]. 

Extracellular Galectin-3 can recognize molecules associated with various pathogens and thus behave as Pattern Recognition Receptor (PRR). PRRs represent a group of receptors which have a role in recognition of microbial “pathogen-associated molecular patterns” (PAMPs), such as constituents of bacterial and fungal cell wall, or viral genome [31,32,33,34]. On the other hand, modulation of innate immune response by intracellular host molecules released from damaged cells upon microorganism-induced necroptosis represent “damage-associated molecular pattern” (DAMP) [31,32,33,34]. 

Examples of PAMP Galectin-3 pathways are bacterial infections in which Galectin-3 recognizes the glycoconjugates of different bacteria, such as *Helicobacter pylori*, *Neisseria meningitidis*, *Neisseria gonorrhoeae*, *Streptococcus pneumoniae*, *Klebsiella pneumonia*, and *Escherichia coli* [31,34,35,36,37]. For instance, in *Helicobacter pylori* infection of the wild type (WT) mice bacterial cells were restricted to the surface of the gastric mucosa, while in the Galectin-3 deficient mice bacterial cells penetrated deep within the gastric glands [38]. Boziki and colleagues reviewed the interaction between *Helicobacter pylori* infection mediated Galectin-3 up-regulation and neurodegeneration [39]. The authors presented various steps in cascade of neurodegeneration such as: common contribution of *Helicobacter pylori* mediators and Galectin-3 in disruption of blood–brain barrier, *Helicobacter pylori* induced increase of Galectin-3 expression in CNS, compromised phagocytic activity of macrophages, triggering production of cytokines involved in pathogenesis of neurodegenerative diseases and other toxic agents [39]. 

Furthermore, Galectin-3 showed direct bacteriostatic properties against *Streptococcus pneumonia* in vitro, while recombinant Galectin-3 decreased severity of pneumonia in Galectin-3 deficient mice [40]. Galectin-3 also has anti-fungal properties in infections caused by *Candida* species [41,42]. Direct cytocidal effect of Galectin-3 on *Candida albicans* is mediated by binding to the beta-1,2-linked oligomannans on the cell surface [42]. 

Galectin-3 as DAMP has role in recruitment and activation of various innate immune cells [34]. In murine model of sepsis induced by *Francisella novicida*, Galectin-3 deficient mice showed reduced inflammatory response and neutrophil accumulation, while in WT mice increased extracellular accumulation of Galectin-3 was followed by hyperinflammatory response [43]. Although Galectin-3 does not take part in chemoattraction of neutrophils [44], as a DAMP it promotes neutrophil migration to inflamed tissues, as well as their removal and termination of inflammatory reaction [31,45].

## 4. Galectin-3 and Matrix Metalloproteinases

It is known that two enzymes, members of the matrix metalloproteinase (MMP) family, MMP-2 and MMP-9 have a role in cleavage of Galectin-3 [46,47]. MMP-2 and MMP-9 catalyze cleavage of Galectin-3 into 22 kDa fragment which contains CRD and 9 kDa polypeptide containing the N-terminal domain. The cleavage 22 kDa fragment has significantly different properties regarding the function and binding properties of CRD [47]. The relationship between Galectin-3 and MMPs has mainly been studied in relation to the processes involved in tumorigenesis and metastasis [48]. The association and positive correlation in Galectin-3 and MMP-1 expression has been demonstrated in highly metastatic melanoma cancer cells (B16F10) [49]. Furthermore, deletion of Galectin-3 resulted in decrease in MMP-1 expression. Increased expression levels of Galectin-3 and MMP-1 were also found in gastric cancer cells, with similar effects of Galectin-3 on MMP-1 [50]. Also, it was shown that Galectin-3 and MMP-9 could be used as a tool for outcome prognosis in patients with hepatocellular carcinoma [51]. The results regarding the effects of recombinant Galectin-3 on keratinocytes indicated the dose-dependent increase of protein and messenger RNA (mRNA) level of MMP-9 [52]. On the other hand, the expression of MMP-9 in recovering epithelium of wounded corneas in C57Bl/6 Galectin-3 KO mice was reduced and was associated with slower healing. There are several studies on the relationship between Galectin-3 and MMPs in nervous tissues. It was shown that MMP-9 deletion had protective effects on ischemic brain injury due to decreased neuroinflammation and preservation of blood–brain barrier [53]. Hypoxic brain injury of Galectin-3-deficient mice indicated decreased expression of MMP-9 in Galectin-3 KO mice in comparison to WT, as well as co-expression of Galectin-3 and MMP-9 in activated microglia [54]. Even though Galectin-3 is a substrate for MMP-9, very little is known about their interaction in nerve structures, both in physiological and pathological processes.

Galectin-3 has numerous pivotal roles in autoimmunity [55,56], kidney disorders [57] immunometabolism, tumor progression [58,59,60,61]. Hara and coworkers reviewed the roles of Galectin-3 in the development of early stages of various diseases [29] In the nervous system different cells types express Galectin-3 in various conditions with versatile, sometimes antagonistic effects. 

## 5. Galectin-3 and Neurodevelopment

Galectin-3 plays an important role in physiological functions of the nervous system, but it is also implicated in variety of neurological disorders. In normal adult rats Galectin-3 is constitutively expressed both in glial cells and neuronal tissues in different brain regions [62]. There is co-expression of Galectin-3 with specific antigens for different cells in rat brain: NeuN (neuronal nuclear antigen), GFAP (glial fibrillary acidic protein), Iba1 (ionized calcium-binding adapter molecule 1). Immunoreactivity for galectin-3 was shown in several regions of telencephalon (some parts of cerebral cortex with variations in the laminar distribution and regions of amygdala, basal ganglia and septum), diencephalon (thalamus and hypothalamus), brain stem and cerebellum (mesencephalon, rhombencephalon, myelencephalon and cerebellum) [62]. Comte and coworkers [63] indicated the role of Galectin-3 in normal neurodevelopment of mouse brain. Galectin-3 affects migration of neuroblasts from subventricular zone (SVZ) through rostral migratory stream (RMS) towards the olfactory bulb (OB) [63]. In Galectin-3 KO mice migration of neuroblasts was disrupted due to the decreased speed and straightness of migration. One of possible mechanisms implies the increased phosphorylation of epidermal growth factor receptor (EGFR) in the absence of Galectin-3 and its increased activation. Keratinocytes with depleted Galectin-3 have impaired migration and decreased surface EGFR expression [64,65].

Galectin-3 has an important role in oligodendrocyte differentiation and maintenance of myelin integrity and function [66]. In vitro study pointed out that oligodendrocytes express Galectin-3 at various stages of differentiation. In vivo experiments also indicated that Galectin-3 mediates oligodendrocyte differentiation by microglial cells and astrocytes [66]. Furthermore, electron microscopic analysis of myelin showed disturbances in myelination process in Galectin-3 KO mice compared to WT. Thomas and Pasquini showed that Galectin-3 mediated glial crosstalk drives oligodendrocytes differentiation [67].

Asphyxia, followed by hypoxic and ischemic brain damage remains one of the most common causes of neurological disorders in infants [68]. Brain damage induced by hypoxia and ischemia results in activation of the immune system and consequent increase in production of pro-inflammatory cytokines and reactive oxygen species [69,70,71]. Microglial cells have the crucial role in this neuroinflammation. They also produce Galectin-3 which has pro-inflammatory effects in both hypoxia and ischemia [72,73]. 

In a study on newborn mice which were subjected to neonatal hypoxia/ischemia, Doverhag and coworkers [54] showed the increased expression of Galectin-3 RNA 8 h, 24 h and 72 h after the injury and Galectin-3 colocalized with Iba-1 in activated microglia close to the injury. Furthermore, Galectin-3-deficient mice were protected compared to their WT littermates, given that neuronal tissue volume loss and regional injury of hippocampus and striatum were significantly reduced in Galectin-3-deficient mice. While there was no statistically significant difference in microglia accumulation between Galectin-3-deficient mice and the WT, there were significantly higher levels of total matrix metalloproteinase 9 (MMP-9) protein levels in WTs suggesting the possibility of modulation of microglia phenotype by Galectin-3 and mechanism of injury attenuation in Galectin-3-deficient mice [54]. Study on C57Bl/6 NADPH oxidase KO mice implicated that hypoxic brain injury increased Galectin-3 levels in NADPH KO mice in comparison to the WT, as well as in injured hemisphere compared to the uninjured hemisphere in both the KO and WT mice [74]. 

Pesheva and collaborators [75] argue that expression of Galectin-3 in neurons depends on the presence of the nerve growth factor (NGF). The authors used neonatal dorsal root ganglion (DRG) neurons to test the effects of NGF, brain-derived neurotrophic factor (BDNF) and neurotrophin-3 on Galectin-3 expression patterns and type of cells which express Galectin-3. They showed that NGF stimulated DRG neurons and macrophage-like cells increased expression of Galectin-3, suggesting a role in promotion of neurite outgrowth and adhesion of neural cells [75,76]. The authors also proposed that molecular mechanism included the activation of TrkA (Tropomyosin receptor kinase A) receptors, while a later study showed that regulation of Galectin-3 expression was mediated through Ras/MAPK-related signaling pathways [75,77]. On the other hand, the same group of authors showed that staurosporine (a protein kinase inhibitor) induced Galectin-3 expression after 1–5 days in culture of PC12 cells which was not affected by Ras/MAPK pathway inhibitors, suggesting also Ras/MAPK-independent mechanism for regulation of Galectin-3 expression [77].

Umekawa and coauthors [78] compared inflammatory responses to brain injury due to hypoxia/ischemia in immature and adult hippocampus, focusing on the differences between resident microglia and macrophages from circulation. Based on the obtained results the authors concluded that in the immature brain resident microglia activated earlier and caused a more pronounced inflammatory response compared to the infiltrating blood-derived macrophages. Furthermore, Galectin-3 expression was more pronounced in immature brains which can be brought into connection with more prominent inflammatory response in newborn animals [78]. Conversely, a study by Chip and coauthors on the role of Galectin-3 in focal stroke caused by transient occlusion of middle cerebral artery resulted in up-regulation of Galectin-3 in neonatal mice and rats [79]. In Galectin-3—deficient mice more severe loss of tissue occurred compared to the wild type [79]. Furthermore, in Galectin-3 deficient mice some of cytokines and chemokines were changed 72 h after the induction of brain damage, where the levels of interleukin 6 (IL-6) and Granulocyte Colony-Stimulating Factor (G-CSF) decreased, and Macrophage Inflammatory Protein 1α (MIP-1α) and MIP-1β increased.

Novel inflammation-independent role of Galectin-3 is shown in regulation of astrogenesis by alteration of bone morphogenetic protein (BMP) signaling [80]. The authors focused on postnatal lateral subventricular zone, given that periventricular regions of the brain are substantially sensitive to hypoxic ischemia, and applied electroporation to increase or decrease Galectin-3 expression in vivo, and nucleofection in vitro [80]. Subventricular zone is the major source of glial cells during postnatal forebrain development, and Galectin-3 deficiency reduces gliogenesis in postnatal period, while Galectin-3 increase has an opposite effect [80]. Furthermore, Galectin-3 binds to bone morphogenetic protein receptor one alpha (BMPR1α), activates BMP signaling and thus regulates basal gliogenesis [80].

## 6. Galectin-3 and Neuroinflammation

Galectin-3 KO mice developed by Hsu and colleagues on C57Bl/6 background are widely used for the evaluation of Galectin-3 role in inflammatory response [59]. They do not appear to have a distinct phenotype compared to the WTs. Behavioral characteristics are described by Stajic and coauthors [81] and discussed further in chapter 9.

There has recently been increasing evidence that Galectin-3 plays a role in neuroinflammation and neurodegeneration [82]. Experimental autoimmune encephalomyelitis (EAE) reflects pathological changes in multiple sclerosis in humans, providing a widely accepted model for this disease [83]. We observed that the deletion of Galectin-3 gene attenuates EAE in C57Bl/6 mice [84]. This was attributed to modulation of antigen-presenting cells and subsequent attenuation of inflammatory response in CNS. Following immunization of WT and Galectin-3 with myelin oligodendrocyte glycoprotein peptide (MOG_35–55_) the severity of diseases was significantly lower in Galectin-3-deficient mice. In addition, production of pro-inflammatory cytokines, IL-17 and IFN-γ, in these mice was reduced, while dendritic cells (DC) produced IL-10 and exhibited Th2 polarization. Galectin-3 is also involved in Interleukin 4 (IL-4) mediated macrophage alternative polarization, thus its effect in EAE may be attributed to its effect on activation and proliferation of microglia [59,84]. Microglia represent immune competent cells in the brain which appear to play the important roles both in diverse homeostatic mechanisms in the nervous tissues and in various pathological conditions which affect brain homeostasis [85]. It has been established that activation of microglia can be a double-edged sword, whereby their profiling depends on many different factors. Reichert and Rotshenker [86] also indicated the role of Galectin-3 in the pathogenesis of EAE. First, they showed increased expression of Galectin-3 in macrophages and microglia in the CNS of mice with EAE. On the other hand, using Copolymer 1 as immunomodulator, they suppressed EAE. This was thought to be due to decreased activation of microglia and macrophages, given that Copolymer 1 induces antigen-specific Th2 response and increased secretion of IL-10, which in turn decreases production of pro-inflammatory cytokines and Galectin-3. It has also been postulated that Galectin-3 may be an important activator of phagocytosis of modified myelin, a necessary stage during its recovery within Wallerian degeneration. There is an increased expression of Galectin-3 in microglia that phagocyte myelin, unlike the microglia that do not phagocyte myelin [87,88,89]. Wallerian degeneration following injury of sciatic nerve in Galectin-3-deficient mice was associated with vigorous increase in inflammatory cytokines, IL-1β and TNF-α, and up-regulation of toll-like receptors (TLR) 2 and 4 [90]. The C57Bl/6 mouse model of focal cortical EAE, which were immunized with MOG and received intracerebral solution of tumor necrosis factor-α (TNF-α) and interferon-γ (IFN-γ) developed large lesions with a high number of Galectin-3-positive inflammatory cells [91]. These cells were classified into two main categories—Galectin-3—positive cells with projections, microglia-derived macrophages, and Galectin-3—positive cells without projections, macrophages derived monocytes. Recent data on cell and stage-specific expression of Galectin-3, in mouse model of EAE induced by pathogenic T-cell transfer, showed increased expression of Galectin-3 in microglia with magnified phagocytic activity in spinal gray matter during progressive disease [92]. Furthermore, the expression of Galectin-3 increased in microglia and macrophages in spinal white matter and pia mater during disease progression, while in nerve roots subpopulation of Schwann cells were Galectin-3—positive. During recovery phase, Galectin-3—expressing cells disappeared from parenchyma, and were confined to the pia mater and ventral nerve roots. These results suggest the possibility of neuroprotective role of Galectin-3 in brain pathology, thus Galectin-3 may have both pro and anti-inflammatory effects in the CNS. Its role appears to depend on the type of cells Galectin-3 expresses and its cellular localization. In addition, the effects of Galectin-3 in type 1 and 2 diabetes can be compared when expressed on immune cells and overexpressed in target cells. We have shown previously that Galectin-3 deletion attenuates type 1 diabetes due to its lack in immune effectors cells [55]. However, in type 2 diabetes intracellular genetic overexpression of Galectin-3 (knock in mice) protects pancreatic β-cells from inflammatory attack [93].

Galectin-3 plays a role in the pathogenesis of viral infections of the CNS. Junin virus-induced encephalitis was induced in C57Bl/6 mice by intracerebral inoculation and it was shown that activated microglial cells and astrocytes express Galectin-3 [94]. In Theiler’s Murine Encephalomyelitis Virus (TMEV) infection Galectin-3 expression increased in the cerebral cortex of in C57Bl/6 and SJL/J mice [95]. Furthermore, Galectin-3 deletion in C57Bl/6 mice reduced the number of activated immune cells after TMEV infection and diminished inflammatory response followed by a partial restoration of SVZ proliferation and increase of SVZ progenitor cells.

## 7. Galectin-3 in the CNS Injury

Galectin-3 appears to be one of the crucial initiators of microglia activation and proliferation following ischemic brain injury, but role of activation of microglial cells upon brain ischemia remains questionable, whether it could be advantageous or injurious [96]. Following experimental stoke in normal C57Bl/6 mice there was strong increase in Galectin-3 expression in microglia surrounding ischemic lesion, while in quiescent microglial cells Galectin-3 immunoreactivity was depressed [72]. In Galectin-3 KO C57Bl/6 mice depletion of Galectin-3 led to inadequate activation and decreased proliferation of microglia, resulting in decrease of microglial cells in ischemic conditions. This disruption in regulation of microglia activity consequently induced increase in infarct size and number of apoptotic neurons. In proliferating microglia there is co-expression of Galectin-3 and Insulin-like growth factor 1 (IGF-1), while in Galectin-3 KO C57Bl/6 mice there increased protein level of IGF-1 after stroke. Specifically, microglia of Galectin-3 depleted mice were not responsive to IGF-1 and it was implicated that Galectin-3 interacted with IGF-1 receptors thus enabling their crosslink at the membrane surface, delay of their removal by endocytosis, and consequently prolonged signaling [64,72]. It has also been shown that Galectin-3 induced proliferation of endothelial cells and neural progenitors upon ischemic brain injury caused by transient middle cerebral artery occlusion (MCAO) in rats, while inhibition of Galectin-3 with anti-Gal-3 antibody had opposite effects [97]. These results suggest a possible role of microglial Galectin-3 in nerve tissue remodeling through angiogenesis and neurogenesis. Recently it was indicated that intracerebroventricular injection of recombinant Galectin-3 during acute phase of stoke, in model of brain ischemia caused by MCAO, induced alternative activation of microglia, increased secretion of IL-4, and decreased production of pro-inflammatory cytokines (TNF-α, IL-1β, INF-γ, IL-6 and IL-17) [98]. Measuring stroke area four days after induction of ischemia and 72 h after the application of recombinant Galectin-3 showed significant reduction in the size of ischemic lesions in mice receiving recombinant Galectin-3 compared to control.

In a study assessing delayed neuronal death induced by transient ischemia in the hippocampal CA1 region, the authors confirmed there was increased expression of Galectin-3 in microglia with the peak which was achieved 96 h following reperfusion [99]. On the other hand, intra-ischemic hypothermia significantly averted delayed neuronal death as well as expression of Galectin-3 in microglial cells. 

Furthermore, investigating the connection between stoke and enteric neuropathy, it was shown that sera from C57BL/6 mice subjected to permanent MCAO induced loss of myenteric neurons in vitro, contrary to sera isolated from Galectin-3—deficient mice with induced stoke [100]. Conversely, myenteric neurons obtained from mice deficient in toll-like receptor 4 (TLR4) were unaffected, and also the application of antagonists of transforming growth factor β-activated kinase 1 (TAK1) or AMP activated kinase (AMPK) prevented loss of myenteric neurons in vitro. Combining the results of this study it can be assumed that the release of Galectin-3 following stroke may induce enteric neuronal cell death via TLR4 activation involving TAK1 and AMPK. 

**Figure 2:**
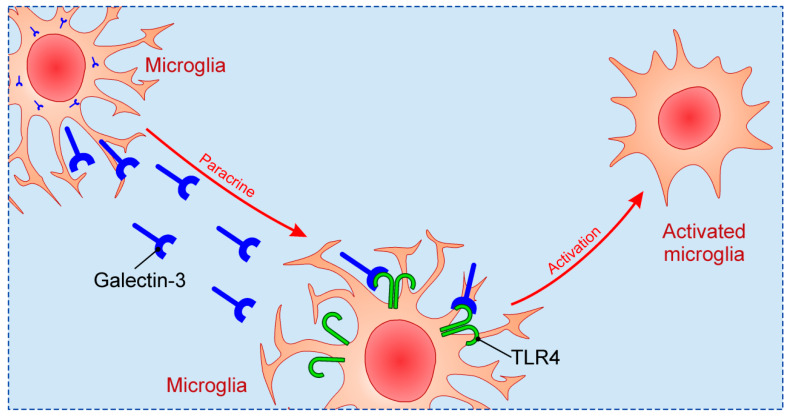
The mechanism of microglial activation by Galectin-3 via TLR4.

Previous studies focused on relationship between Galectin-3 and TLR family in CNS, in particular TLR2 and TLR4. Galectin-3 possesses great affinity for β-galactoside, and considering that TLR4 structurally contains β-galactosides, Galectin-3 binding to TLR4 occurs [101]. Such binding can induce changes in TLR4 appearance, for instance dimerization or internalization. Inflammatory stimulation in brain, such as intranigral injection of lipopolysaccharide (LPS), triggers endogenous production of Galectin-3, which binds to microglial TLR4 and causes their activation [102] (Figure 2). On the other hand, intranigral LPS injection and consequent neuroinflammation in Galectin-3 KO mice were characterized by reduced expression of pro-inflammatory markers, IL-1β and IL-6, and decreased inflammatory response with neuroprotective effect. Effects of in vitro exposition of microglial cells to soluble Galectin-3 were increase of expression of inducible nitric oxide synthase (iNOS) and stimulation of pro-inflammatory M1 phenotype combined with decrease of anti-inflammatory markers. Expression of TLR4 and Galectin-3 were also increased in burn-induced peripheral neuroinflammation [103]. TLR4 appears to be universal receptor for Galectin-3 in inflammation in different tissues [104]. Galectin-3 seems to be an important mediator in injury-induced innate immune response/TLR2 signaling [72]. In microglial cells of Galectin-3 KO mice there was no up-regulation of TLR2 due to stimulation with glutamate, contrary to WT cells. It has also been shown that LPS induction of Galectin-3 release from microglia enhances microglial phagocytosis [105]. Specifically, secreted Galectin-3 opsonizes cells for phagocytosis via interaction with a phagocytic receptor, Mer tyrosine kinase (MerTK), and this process appears to be necessary for phagocytosis of cells by activated microglia [106].

Traumatic injury of CNS that encompasses devastation of various neuronal structures, including axonal damage and destruction of myelin, is followed by infiltration of damaged tissue with immune cells and consequent activation of microglia, which precedes regeneration and scarring. In mouse model of cortical contusion, expression of Galectin-3 significantly increased after impact in cortex and hippocampus, followed by increased levels of Galectin-3 in cerebrospinal fluid, and interaction between Galectin-3 and TLR4 [107]. Application of anti-Gal-3 antibody exhibited neuroprotective effect due to decreased trauma-induced synthesis of pro-inflammatory markers, IL-1β, IL-6 and iNOS, and attenuated microglial activation. Conversely, Galectin-3 deletion was unsuccessful in modulation of traumatic brain injury (TBI) induced pro-inflammatory response, suggesting the importance of difference in relation to complete, constitutive absence of Galectin-3 and antagonizing only extracellular Galectin-3. Clinical study on Galectin-3 as a possible prognostic marker in patients with severe TBI showed significant increase of plasma Galectin-3 in TBI patients. The value of Galectin-3 positively correlated with severity noted by Glasgow Coma Scale scores and levels of plasma C-reactive protein [108]. Increase of plasma Galectin-3 was also shown in patients with mild TBI by others [109].

Spinal cord injury (SCI) is also followed by neuroinflammation with significant and prolonged expression of pro-inflammatory proteins, including Galectin-3 [110,111]. Confirming these results, Galectin-3-deficient mice had better functional recovery after SCI due to limitation of lesions without spreading to the surrounding area and maintenance of white matter integrity [112]. With respect to inflammatory response there was a decrease of CD11b-positive cells in Galectin-3-deficient mice, associated with increase of Arginase1-positive cells. Specifically, Arginase1-expressing cells represent anti-inflammatory M2 macrophages characterized by production of anti-inflammatory cytokines [113].

## 8. Galectin-3 and the Mechanism of Neurodegeneration

### 8.1. Prion Disease

Mok and coauthors [114] suggested a detrimental role of Galectin-3 in prion diseases. They identified microglia as the major cell type expressing Galectin-3. Ablation of Galectin-3 did not affect the abnormally folded prion protein (PrpSc) and development of gliosis. However, Galectin-3 deficient mice showed reduced level of lysosomal activation marker LAMP-2 (lysosome-associated membrane protein 2) and prolonged survival time.

### 8.2. Alzheimer’s Disease

It has been recently shown that Galectin-3 appears to be involved in inflammatory response in neurodegenerative disorders such as Alzheimer’s disease, Parkinson’s disease, and Huntington’s disease. Alzheimer’s disease (AD) is a progressive degenerative disorder featured by the extraneuronal accumulation of the amyloid β (Aβ) protein in the form of plaques and the intraneuronal aggregation of the microtubule-associated protein tau in the form of filaments [115]. Galectin-3 is highly up-regulated in brain cortex and glial cells in AD patients compared to age-matched healthy controls [115,116]. In experimental model of familial AD using 5×FAD mice showed very early appearance of microglial cells which express Galectin-3. Furthermore, Galectin-3 appears to be a very important inflammatory mediator in AD, given that reduction of Galectin-3 led to a significant drop of pro-inflammatory cytokines (IL6, IL8 and TNFα), while the absence of Galectin-3 in 5×FAD mice reduced pathological changes [116]. Molecular mechanisms which mediate the switch from resident, homeostatic microglia towards disease-associated microglia include up-regulation of TREM2 (Triggering Receptor Expressed on Myeloid cells 2). Intra-hippocampal injection of Aβ in C57Bl/6 WT mice triggers Aβ oligomerization, but in Galectin-3-deficient C57Bl/6 mice Aβ oligomerization is reduced [117]. Assessing the APP/PS1 mice (double transgenic mice expressing a chimeric mouse/human amyloid precursor protein and a mutant human presenilin 1) it was shown that endogenous Aβ oligomerization and expression of Galectin-3 are increased in an age-dependent manner, while in APP/PS1;Gal3^+/−^ Galectin-3 level was significantly lower. Also, protein inhibitor of activated STAT1 (Signal Transducer and Activator of Transcription 1), which plays an important role in regulation of innate immune responses and has anti-inflammatory properties, decreased in aged APP/PS1 mice. 

### 8.3. Parkinson’s Disease

Parkinson’s disease (PD) is a progressive neurodegenerative disease characterized by pathological changes that encompass formation of α-synuclein aggregate accumulation known as Lewy bodies and progressive loss or deregulation of dopaminergic neurons in the substantia nigra of the midbrain, followed by glial activation and neuroinflammation [118]. Following treatment of microglial cells with recombinant α-synuclein increased expression of iNOS was observed indicating activation of microglia, which was followed by up-regulation of pro-inflammatory cytokines (TNF-α, IL-2 and IL-12) [119]. Simultaneous pharmacological inhibition of Galectin-3 and genetic deletion of Galectin-3 induced inhibition of α-synuclein-mediated activation of microglia and decrease of pro-inflammatory cytokine production. In vivo application of α-synuclein in olfactory bulb of WT mice showed that upon taking up α-synuclein microglia exert Galectin-3-positive phenotype. Clinical investigation pointed out raised levels of serum Galectin-3 in patients with idiopathic PD, suggesting importance of Galectin-3 in pathogenesis and possible prediction of PD implying a role of Galectin-3 as a biomarker for PD detection, prediction of disease severity and disease prevention [120].

### 8.4. Huntington’s Disease

It appears that Galectin-3 also plays a role in brain inflammation associated with Huntington’s disease (HD) [121]. Siew and collaborators showed that plasma Galectin-3 was higher in HD patients compared to healthy controls, and importantly plasma levels of Galectin-3 significantly correlated with severity of the disease [121]. Post-mortem brain analysis of HD patients indicated up-regulation of Galectin-3 in the caudate putamen region of the brain. The experiments conducted in R6/2 mice, transgenic mouse model of HD which expresses mutant huntingtin gene (mHTT), pointed out increased Galectin-3 level in the striatum of R6/2 compared to WT mice. Nuclear factor κB (NFκB) seems to be an important mediator of Galectin-3 regulation in HD considering that inhibition of NFκB induces decrease in microglia activation and decrease in production of pro-inflammatory cytokines. Galectin-3 also increased production of inflammatory cytokines by HD microglia [121].

## 9. Galectin-3 and Behavior 

Evidence is emerging that Galectin-3 may also play a role in behavioral pattern of mice and may be involved in the various forms of behavioral alterations, such as mood disorders (anxiety and depression), cognitive dysfunctions, attention deficits, and psychosis. 

By means of the experimental design this has been mainly studied in Galectin-3—deficient mice. 

Deletion of Galectin-3 affects baseline behavioral patterns in healthy mice compared to wild type age-matched controls [122]. Assessing patterns of feeding, drinking, movement, and circadian rhythm in Galectin-3 KO mice, a decrease in locomotor activity, especially during the dark phase of circadian cycle was observed, with no overall difference in food or water intake. Interestingly, these differences were more pronounced in aged mice (6–7 months old) compared to younger animals (2–3 months old). This observation may be considered to be a supporting evidence for the role of Galectin-3 in development and maturation of certain regions of CNS. Furthermore, the analysis of multiple behavioral test parameters showed differences between Galectin-3—deficient and WT mice in two assays, the single-cue aspect of the conditioned fear task and the test of social dominance [81]. Galectin-3 KO C57BL/6 mice had lower ability to become immobile when exposed to conditioned stimulus in comparison to WT mice [81]. The social dominance test indicated domination of Galectin-3 KO mice compared to WT, whereby Galectin-3 KO mice displayed aggressive behavior.

The clinical trial assessing association between genetic variations in the gene encoding Galectin-3 and cognitive impairment in the elderly population showed significant correlation between variant alleles and decline in cognitive performance [123]. Despite the lack of data on serum Galectin-3, the results of this study indicated that all subjects with *LGALS3* gene variations had increased C-reactive protein, suggesting that inflammation as a possible mechanism of Galectin-3 mediated on cognitive dysfunction.

**Figure 3:**
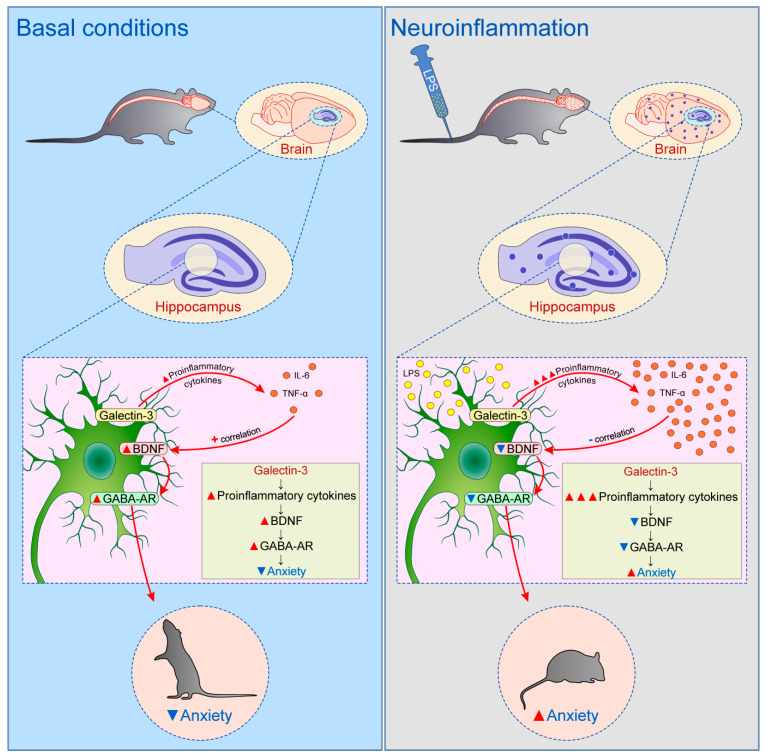
The postulated mechanism of Galectin-3 role in anxiety level regulation in mice hippocampus—the alterations depending on basal vs. neuroinflammatory conditions.

Most recently we evaluated the role of Galectin-3 deficiency on anxiety levels in C57Bl/6 mice, under physiological conditions, as well as following acute LPS-induced neuroinflammation [124]. The major finding was the observation of the two contradictory effects of Galectin-3 on anxiety level depending on the animals’ preconditioning. Specifically, in untreated animals the existence of Galectin-3 in hippocampus resulted in anxiolytic effect, while the Galectin-3 expression in mice hippocampus was accompanied by enhancement of anxiogenic response following acute LPS-induced neuroinflammation. Unsurprisingly, WT animals showed oversized anxiogenic-like behavior, when compared to Galectin-3 KO animals, during the early phase (24 h) of neuroinflammation. The anxiogenic response to intact Galectin-3 content was concomitant with the enhanced pro-inflammatory cytokines (IL-6 and TNF-α release) which negatively correlated with hippocampal BDNF content that was accompanied by GABA-A receptors decline in hippocampus, finally resulting in anxiety-like behavior (Figure 3). Even more interesting, the anxiolytic impact of Galectin-3 under physiological conditions was confirmed by means of the principle anxiety level indicators obtained in various behavioral tests, and supported by additional data based on the alterations in evaluated locomotion patterns, as well as an exploratory activity behavior [125]. The results of this study also showed that the beneficial effect of Galectin-3 on mood regulation (expressed by anxiolytic response) occurred simultaneously with the increased hippocampal expression of the cytokines, although less prominently than during the neuroinflammation. However, under basal conditions, hippocampal cytokines augmentation positively correlated with the BDNF content, unlike during the acute phase of the neuroinflammation. As expected, this sequence of events was accompanied by the positive impact on hippocampal GABAergic system and consequent anxiolytic effect (Figure 3). Beside this causal evaluation of the Galectin-3 role in anxiety level regulation, focused on the confirmed mechanisms involved in mood control, there was evidence that Galectin-3 may also induce anxiolytic effect by its overall impact on the myelination process and myelin sustention [66], estimated in the cuprizone model of demyelination [125].

Galectin-3 appears to play one of the main roles in neuroinflammation associated with the pathogenesis of AD. It has been shown that stereotaxic administration of Aβ_25–35_ into CA1 region of rat hippocampus induced inflammation and increased expression of Galectin-3 accompanied by the loss of spatial memory [126]. Furthermore, cognitive performance was improved by Galectin-3 gene deletion (APP/PS1; Gal-3^+/−^ mice) in APP/PS1 mice, prone to AD [117]. In line with this, the clinical trials also pointed out that AD patients have increased expression of Galectin-3 in frontal lobe, while the serum Galectin-3 levels positively correlated with the severity of memory loss [117]. 

Although the significance of inflammation in the pathogenesis of schizophrenia is well documented, the role of Galectin-3 in this process remains unclear [127]. Galectin-3 serum levels in patients with schizophrenia were higher compared to healthy controls and positively correlated with the results obtained in the Brief Psychiatric Rating Scale [128]. An investigation regarding the interconnections between Galectin-3, IL-33 and soluble ST2 (sST2) in various stages of schizophrenia showed significant alterations of these markers of innate immunity in disease remission and exacerbation [129]. 

Depression may be related to neuroinflammation, considering that immune response may affect neurotransmission [130]. Patients suffering from depression associated with type 1 diabetes showed higher levels of plasma Galectin-3, which was not associated with other commonly measured variables in diabetic patients, suggesting that Galectin-3 could contribute to development of AD, cardiovascular complications or cause mortality in depressed persons [131]. 

In addition, the majority of data considering a potential role of Galectin-3 in attention deficit hyperactivity disorder (ADHD) was obtained due to fact that the spontaneously hypertensive rats (SHR) were found to be an experimental model of ADHD [132]. Decreased expression of Galectin-3 in brains of juvenile 6-week-old SHR [133] was observed in prefrontal cortex, striatum, and midbrain. This was accompanied by the alterations in the expression of tyrosine hydroxylase and dopamine synthesis. Accompanied by the reported decreased circulating Galectin-3 levels in children with ADHD syndrome, combined with increased circulating let-7d miRNA [134], it appears that Galectin-3 may also have a significant role in the pathogenesis of ADHD syndrome.

## 10. Inhibitors of Galectin-3 and Their Potential Therapeutic Use

Recent studies dealing with Galectin-3 inhibitors suggest their potential use in various pathological conditions involving this protein in their pathogenesis. Considering the various localization of Galectin-3 in the tissue (intracellular, membrane-bound or extracellular), as well as its diverse effects, different antagonists have been synthesized with variations in cellular uptake [135,136]. Small molecule Galectin-3 inhibitor, 33DFTG, diminished pathological corneal neovascularization and fibrosis in animal models [137]. Another antifibrotic effect of use of the Galectin-3 inhibitors was shown in murine model of lung fibrosis induced by bleomycin [138]. Furthermore, bleomycin-induced lung fibrosis in Galectin-3—deficient mice was significantly diminished due to reduced transforming growth factor (TGF)-β regulated myofibroblast activation and production of collagen. Galectin-3 inhibitor, TD139, also impaired progression of lung fibrosis [139]. Using model of concanavalin A-induced liver injury, it has been shown that pretreatment with selective Galectin-3 inhibitor, TD139, induces blander liver infiltration with pro-inflammatory CD4(+) T cells and pronounced presence of anti-inflammatory cells, resulting in attenuation of liver injury [140]. TD139 also decreased production of pro-inflammatory cytokines (TNF-α, IL-12, IFN-γ) by liver dendritic cells (DCs) and Natural killer T (NKT) cells in α-galactosylceramide induced hepatitis [141]. Furthermore, selective inhibition of Galectin-3 decreased liver infiltration with pro-inflammatory CD11c+CD11b+ DCs, and increased production of IL-10 by liver DCs and NKT cells [141]. In papillary thyroid cancer cell lines application of Galectin-3 inhibitor was followed by enhanced apoptosis, hemosensitivity, and radiosensitivity [142]. One of the few neuropathological studies addressing the role of Galectin-3 inhibitors in the brain structure showed that modified citrus pectin (Galectin-3 inhibitor) prevents breakdown of blood–brain barrier and brain injury in mouse model of subarachnoid hemorrhage [143]. These results point out the possibility of addressing Galectin-3 as a therapeutic target in a variety of pathological conditions as an immunomodulator. 

## 11. Conclusions

This review summarizes the roles of Galectin-3 in cellular pathology and discusses in some detail our knowledge of its roles in neuroinflammation, neurodegeneration, and possible effects on cognitive functions. Multiple and sometimes contrasting functions of Galectin-3 stem from the expression of this protein in the nucleus, cytoplasm, mitochondria, cell surface, and extracellular matrix. In neuropathology, cell surface, and extracellular Galectin-3 appear to be most important.

In animal model studies of the Galectin-3 roles, several research groups used Galectin-3 KO mice on C57Bl/6 background. 

It was established that inflammatory T-cell mediated autoimmunity in hepatitis, pancreatitis, myocarditis, and type 1 diabetes mellitus, Galectin-3 had strong pro-inflammatory effects. We then analyzed the role of Galectin-3 in neuroinflammatory, neurodegenerative conditions, and behavioral alteration. Similar to other T-cell mediated diseases deletion of Galectin-3 suppresses the induction EAE. Data obtained by others and ourselves clearly demonstrated that Galectin-3-TLR4 interaction is required for induction of neuroinflammation.

The investigations of the roles of Galectin-3 in neurodegenerative diseases are still in nascent phase. 

Early studies in mouse prion disease showed that lack of Galectin-3 attenuate this condition. More importantly, in Alzheimer’s disease Galectin-3 appear to be one of the key molecules in microglial activation and Galectin-3 deletion in AD prone mice attenuate microglia associated TLR dependent immune response, amyloid β burden and improves cognitive functions. 

Finally, Galectin-3 appears to have a very complex impact on various behavioral patterns. Some of them strongly depend on age-dependent characteristics, such as maturation and development of certain brain regions, such as cognitive functions and response to stress. On the other hand, the final conclusion considering the anxiety level control influenced by Galectin-3 is crucially affected by the existence of inflammation in the targeted tissue. Taking into account data obtained in behavioral investigations it seems that Galectin-3 role in control of behavioral patterns should be extensively evaluated with the special referring to the influence of various variables that may substantially define its final effect.

It is involved in maturation as well as in behavioral response in neuroinflammation. In summing up, Galectin-3 may act as a ligand and a receptor in cell to cell interaction. It is primarily a pro-inflammatory factor and appears to be a valid biomarker for detection and evaluation of disease progression. 

In the CNS Galectin-3 is multifunctional, primarily affects microglia and there is interaction with the resident and invading cells. Very recent data reveal that studies of Galectin-3 in the CNS contribute to a better understanding of the pathology of neurodegeneration and behavioral dysfunction.

Finally, the recently developed highly selective inhibitors of extra and intracellular Galectin-3 will provide new approaches in this field and potentially define Galectin-3 as a therapeutic target.
